# C_60_ fullerene attenuates muscle force reduction in a rat during fatigue development

**DOI:** 10.1016/j.heliyon.2022.e12449

**Published:** 2022-12-21

**Authors:** Yuriy Prylutskyy, Dmytro Nozdrenko, Olga Gonchar, Svitlana Prylutska, Kateryna Bogutska, Daria Franskevych, Bohdan Hromovyk, Peter Scharff, Uwe Ritter

**Affiliations:** a Department of Biophysics and Medical Informatics, ESC “Institute of Biology and Medicine”, Taras Shevchenko National University of Kyiv, Kyiv 01601, Ukraine; b Hypoxia Department, Bogomoletz Institute of Physiology, NAS of Ukraine, Kyiv 01024, Ukraine; c Department of Physiology, Plant Biochemistry and Bioenergetics, Faculty of Plant Protection, Biotechnology and Ecology, National University of Life and Environmental Science of Ukraine, Kyiv 03041, Ukraine; d Faculty of Pharmacy, Danylo Halytsky Lviv National Medical University, Lviv 79010, Ukraine; e Institute of Chemistry and Biotechnology, Technical University of Ilmenau, Ilmenau, 98693, Germany

**Keywords:** С_60_ fullerene, N-acetylcysteine, *m. soleus*, *m. gastrocnemius*, Muscle fatigue, Biomechanical and biochemical parameters

## Abstract

C_60_ fullerene (C_60_) as a nanocarbon particle, compatible with biological structures, capable of penetrating through cell membranes and effectively scavenging free radicals, is widely used in biomedicine. A protective effect of C_60_ on the biomechanics of fast (*m. gastrocnemius*) and slow (*m. soleus*) muscle contraction in rats and the pro- and antioxidant balance of muscle tissue during the development of muscle fatigue was studied compared to the same effect of the known antioxidant N-acetylcysteine (NAC). C_60_ and NAC were administered intraperitoneally at doses of 1 and 150 mg kg^−1^, respectively, daily for 5 days and 1 h before the start of the experiment. The following quantitative markers of muscle fatigue were used: the force of muscle contraction, the level of accumulation of secondary products of lipid peroxidation (TBARS) and the oxygen metabolite H_2_O_2_, the activity of first-line antioxidant defense enzymes (superoxide dismutase (SOD) and catalase (CAT)), and the condition of the glutathione system (reduced glutathione (GSH) content and the activity of the glutathione peroxidase (GP_x_) enzyme). The analysis of the muscle contraction force dynamics in rats against the background of induced muscle fatigue showed, that the effect of C_60_, 1 h after drug administration, was (15–17)% more effective on fast muscles than on slow muscles. A further slight increase in the effect of C_60_ was revealed after 2 h of drug injection, (7–9)% in the case of *m. gastrocnemius* and (5–6)% in the case of *m. soleus*. An increase in the effect of using C_60_ occurred within 4 days (the difference between 4 and 5 days did not exceed (3–5)%) and exceeded the effect of NAC by (32–34)%. The analysis of biochemical parameters in rat muscle tissues showed that long-term application of C_60_ contributed to their decrease by (10–30)% and (5–20)% in fast and slow muscles, respectively, on the 5th day of the experiment. At the same time, the protective effect of C_60_ was higher compared to NAC by (28–44)%. The obtained results indicate the prospect of using C_60_ as a potential protective nano agent to improve the efficiency of skeletal muscle function by modifying the reactive oxygen species-dependent mechanisms that play an important role in the processes of muscle fatigue development.

## 1 Introduction

Today, the term “muscle fatigue” refers to a wide range of dysfunctions, namely: physiological, neurological, and psychiatric [1, 2]. Therefore, there are diverse concepts about ways of muscle fatigue development [3], the existence of which is because there is no separate mechanism for the complex process of fatigue development. However, it involves a whole complex of mechanisms of central nervous system dysfunction, peripheral nerve dysfunctions, and muscles themselves. All mechanisms are united by the result of functional changes caused by them, mostly by the impossibility of maintaining the required level of effort by the muscle during contraction [4]. Muscle fatigue is a protective mechanism of the body against overloads in general and further development of pain sensitivity of muscles [5]. Its nature and optimal degree are key factors for forming adaptation and increasing the level of functional and physical capabilities of the body. It has been shown that during intense physical load, the duration of recovery periods (active rest) is quite important for maintaining optimal performance and a normal physiological state of actively contracting muscles [6].

During the development of fatigue, there is a slowdown in force generation and muscle relaxation [7]. Skeletal muscle fibers continuously generate reactive oxygen species (ROS) at a slow rate, which increases during muscle contraction. This activity-dependent increase in ROS production contributes to skeletal muscle fatigue during strenuous exercise. Experimental evidence suggests that ROS of muscle origin primarily act on myofibrillar proteins, suppressing calcium sensitivity and reducing contraction force [8]. More intensive exposure to ROS leads to losses in calcium regulation, which mimic pathological changes and are irreversible.

Oxidative stress is a generally recognized pathophysiological factor in the formation of muscle fatigue and overexertion under conditions of excessive physical activity [9]. There are several sources of ROS production in skeletal muscles that are activated during muscle contraction: mitochondrial respiratory chain, lipoxygenase pathway of arachidonic acid metabolism, NADPH-oxidase, and xanthine oxidase [10, 11]. Superoxide, hydroxyl radicals, and hydrogen peroxide are considered to be the most reactive oxygen derivatives that can be formed during the development of muscle fatigue in enzymatic and non-enzymatic reactions [12]. It is known that pathological effects in muscle tissue arise due to excessive accumulation of ROS, peroxides, and their secondary products with the inability of the antioxidant system to ensure the maintenance of prooxidant-antioxidant balance [13]. The functional basis of the antioxidant defense system is formed by the glutathione system, the components of which are glutathione and enzymes that catalyze the reactions of reverse transformation (oxidation or recovery) [14].

During the intensive physical activity of skeletal muscle, lipid peroxidation (LPO) is the primary reaction in the chain of physicochemical transformations that lead to the destruction of the lipoprotein complex of myocyte membranes, disruption of their transport functions, inhibition of oxidative phosphorylation processes, and energy generation, which ultimately reduces cell viability and contributes to muscle fatigue [15]. However, the dynamics of lipoperoxidation product accumulation and the duration of such changes depend on many factors, one of which is the type of muscle fibers, because the latter are characterized by specific metabolic reactions and antioxidant protection features [12]. It is known that there are two types of fibers in the muscle structure: slow and fast-contracting ones. These fibers are different independent functional units distinguished not only by contractile but also by morphological and biochemical properties [16].

Biocompatible carbon nanostructures can be considered potential antioxidants to affect the muscular system, some of which are C_60_ fullerenes [17, 18]. C_60_ molecule is a rather powerful electron acceptor, capable of adding up to six electrons. It is the double chemical bonds on the almost spherical surface of C_60_ fullerene that are electron-deficient ones, that determine its ability to effectively capture free radicals [19, 20]. Earlier in *in vivo* experiments, we tested the antioxidant properties of C_60_ fullerenes about the development of some muscle pathologies [21]. Thus, we can assume the correction of muscle fatigue processes due to the protective effect of C_60_ fullerene, as a powerful antioxidant, on the contractile muscular apparatus.

Here, we studied the antioxidant effect of C_60_ fullerene on the biomechanics of fast (*m. gastrocnemius*) and slow (*m. soleus*) muscle contraction in rats, the pro- and antioxidant balance of their body during the development of muscle fatigue compared to the effect of the known antioxidant N-acetylcysteine (NAC) [22].

## 2 Materials and methods

### 2.1 Preparation and characterization of C_60_FAS

To obtain C_60_ fullerene aqueous solution (C_60_FAS), a method based on the transfer of C_60_ molecules (Sigma Cat. No. 379646) from toluene into the water followed by ultrasound treatment was applied [23, 24]. The mechanism of C_60_ molecule dispersal in an aqueous solution could be explained by a formation of a covalent bond between hydroxyls and carbons in the C_60_ fullerene cage as a result of ultrasound treatment, which culminates in consequent easy C_60_ molecule dissolution [25]*.* The obtained C_60_FAS at a maximum concentration of 0.15 mg mL^−1^ was stable for 18 months at +4 °C.

The structural state of C_60_FAS was studied by the atomic force microscopy (AFM) technique [26]. To do this, a drop of C_60_FAS was applied to the atomically smooth surface of the substrate, and the measurements were carried out after the complete evaporation of water. Freshly cleaved mica surface (muscovite, grade V1) was used as a substrate for AFM research. Measurements were carried out on the system “Solver Pro M” (NT-MDT, Russia) in tapping mode using AFM probes RTESPA-150 (Bruker, USA).

### 2.2 Animals

Male Wistar rats (170 ± 12 g, 2-month-old) were bred and housed in standard temperature conditions (21–23 °C), lighting (12/12 h light-dark cycle), at humidity (30–35%). All animals had unlimited access to chow and tap water. The study was carried out in strict accordance with the European convention for the protection of vertebrate animals used for experimental and other scientific purposes (Strasbourg, 1986) and was approved by the Bioethical Committee of the ESC “Institute of Biology and Medicine” of the Taras Shevchenko National University of Kyiv, Ukraine (ethic code: No. 3447–IV 21.02.2006).

The following groups of animals were tested: experimental groups - after 1,2,3,4 and 5 days of C_60_ fullerene (*n* = 7) and NAC (*n* = 7) administration, respectively, which were compared with the control (“fatigue”, saline administration) (*n* = 7) and intact (“norm”, no fatigue) (*n* = 7) groups.

The research protocol involved intraperitoneal injection of C_60_ fullerene and NAC at a daily dose of 1 and 150 mg kg^−1^, respectively, 1 h before the experiment for 5 days. The choice of the most optimal dose of the applied drugs, showing a positive protective effect on the development of fatigue processes in the skeletal muscle, is due to the results of our previous studies [27, 40]. An increase/decrease in the range of tested doses did not reveal significant changes in muscle dynamics and, in our opinion, the doses used are optimal.

It is important to note that the selected dose of water-soluble C_60_ fullerene in our experiments is significantly lower than the LD_50_ value, which was 600 mg kg^−1^ body weight when administered orally to rats [19] and 721 mg kg^−1^ when administered intraperitoneally to mice [28].

### 2.3 Biomechanical analysis

The object of the study was the slow muscle, *m. soleus*, and fast muscle, *m. gastrocnemius.*

Animals under deep anesthesia (ketamine (100 mg kg^−1^, Pfizer, USA) combined with xylazine (10 mg kg^−1^, Interchemie, Holland)) underwent a tracheotomy and were connected to an artificial lung ventilator. Then the corresponding muscles were dissected, carefully isolating them from the surrounding tissues. The animal was fixed in a stereotaxic machine with a system of rigid fixation of the head, pelvis, and limbs. The isolated nerve was fixed on a bipolar platinum wire electrode for further electrical stimulation. The edges of the skin on the hind limbs around the incision were sutured to the armature of the machine, and the formed trays with muscle and nerve were filled with vaseline oil. During the operation and the experiment itself, the heart rate was monitored. If necessary, a mixture of physiological saline, rheopolyglucin, and glucose was administered, and anesthesia was continued by intraperitoneal administration of a mixture of ketamine/xylazine (¼ of the initial dose) every 30–40 min until the end of the experiment. Body and oil temperatures were maintained at 37–38 °C with an infrared lamp.

The muscle was connected via the Achilles tendon to a servo-control muscle puller. A linear motor under position servo-control was used as a muscle puller. The muscle tension was measured by semi-conductor strain gauge resistors glued on a stiff steel beam mounted on the moving part of a linear motor. The puller’s stiffness exceeded 0.06 N mm^−1^, while the time constants of the length transients did not exceed 60 ms.

Muscle fatigue was induced by successive stimulation impulses with a frequency of 50 Hz and a duration of 5 s each, without a relaxation period between them. The sum of such stimulation signals was 500 s, followed by 5 min of relaxation. The number of stimulation pools was three. Each series of stimulation consisted of separate series of rectangular 2 ms impulses. The current strength at which the muscle began to contract was considered the threshold, further stimulation was performed with a force of 1.3–1.4 of the threshold [29].

During the analysis of the results, we used quantitative parameters – integrated muscle power (calculated area under the strength curve), which is an indicator of its general performance under the applied stimulation pools [30].

### 2.4 Biochemical analysis

LPO was measured from the formation of thiobarbituric acid-reactive substances (TBARS) using the method [31]. TBARS were isolated by boiling tissue homogenates for 15 min at 100 °C with a thiobarbituric acid reagent and measuring the absorbance at 532 nm. The results were expressed as nM mg^−1^ of protein using ϵ = 1.56 × 10^5^ mmol^−1^ cm^−1^.

The H_2_O_2_ concentration in the tissue homogenates was measured using the method, which is based on the peroxide-mediated oxidation of Fe^2+^, followed by the reaction of Fe^3+^ with xylenol orange (o-cresolsulphonephthalein 3′,3″-bis[methylimino] diacetic acid, sodium salt). This method is extremely sensitive and is used to measure low levels of water-soluble hydroperoxide present in the aqueous phase. To determine the H_2_O_2_ concentration, 500 μL of the incubation medium was added to 500 μL of an assay reagent. The absorbance of the Fe^3+^-xylenol orange complex (A560) was detected after 45 min. Standard curves of H_2_O_2_ were obtained for each independent experiment by adding variable amounts of H_2_O_2_ to 500 μL of basal medium mixed with 500 μL of an assay reagent. Data were normalized and expressed as μM H_2_O_2_ per mg protein [32].

Measurements of TBARS adducts provide unambiguous evidence for LPO, and an increase in the abundance of these adducts is likely to be reflective of increased oxidative stress. However, these assays are unspecific since TBA generates chromogens from many biomolecules other than MDA, making quantification of the total extent of LPO somewhat challenging [33]. Therefore, to obtain a complete picture of the dynamics of the oxidative processes, we additionally measured select robust indices to assess the ratio of ROS generation/removal such as the activity of antiradical and anti-peroxide enzymes (superoxide dismutase (SOD), catalase (CAT), selenium-dependent glutathione peroxidase (GP_x_)) and reduced glutathione (GSH) level.

Total SOD activity was measured by the method [34], which is based on the inhibition of autooxidation of adrenaline to adrenochrome by SOD contained in the examined samples. The results were expressed as specific activity of the enzyme in units per mg protein. One unit of SOD activity is defined as the amount of protein, causing 50% inhibition of the conversion rate of adrenaline to adrenochrome, under specified conditions.

CAT activity was measured by the decomposition of hydrogen peroxide, determined by a decrease in the absorbance at 240 nm [35].

A GP_x_ activity was determined according to the method [36]. The rate of NADPH oxidation followed at 340 nm.

The GSH level was determined as described in [37]. The tissue sample was mixed with sulphosalicylic acid (4%) and incubated at 4 °C for 30 min. Thereafter, it was centrifuged at 1200 × g for 15 min at 4 °C and 0.1 mL of this supernatant was added to phosphate buffer. The yellow color developed was read immediately at 412 nm. The GSH content was calculated as mmol L^−1^ mg^−1^ protein (ε_412_ = 13.6 × 10^3^ mol^−1^ cm^−1^).

### 2.5 Statistical analysis

Statistical processing of the measurement results was performed by methods of variational statistics using the software Origin 9.4. Each of the experimental force curves obtained in the work is the result of averaging 10 similar experiments. At least three repetitions were performed for each biochemical measurement. Data are expressed as the means ± SEM for each group. The differences among experimental groups were detected by one-way ANOVA followed by Bonferroni’s multiple comparison test. Values of *p* < 0.05 were considered significant.

## 3 Results and discussion

### 3.1 AFM analysis of C_60_FAS

The biological activity of pristine C_60_ fullerene largely depends on its concentration in the aqueous medium, and the size distribution of the formed nanoparticles, which, in particular, explains some inconsistencies in the toxicity of C_60_ fullerene [38, 39].

**Figure 1:**
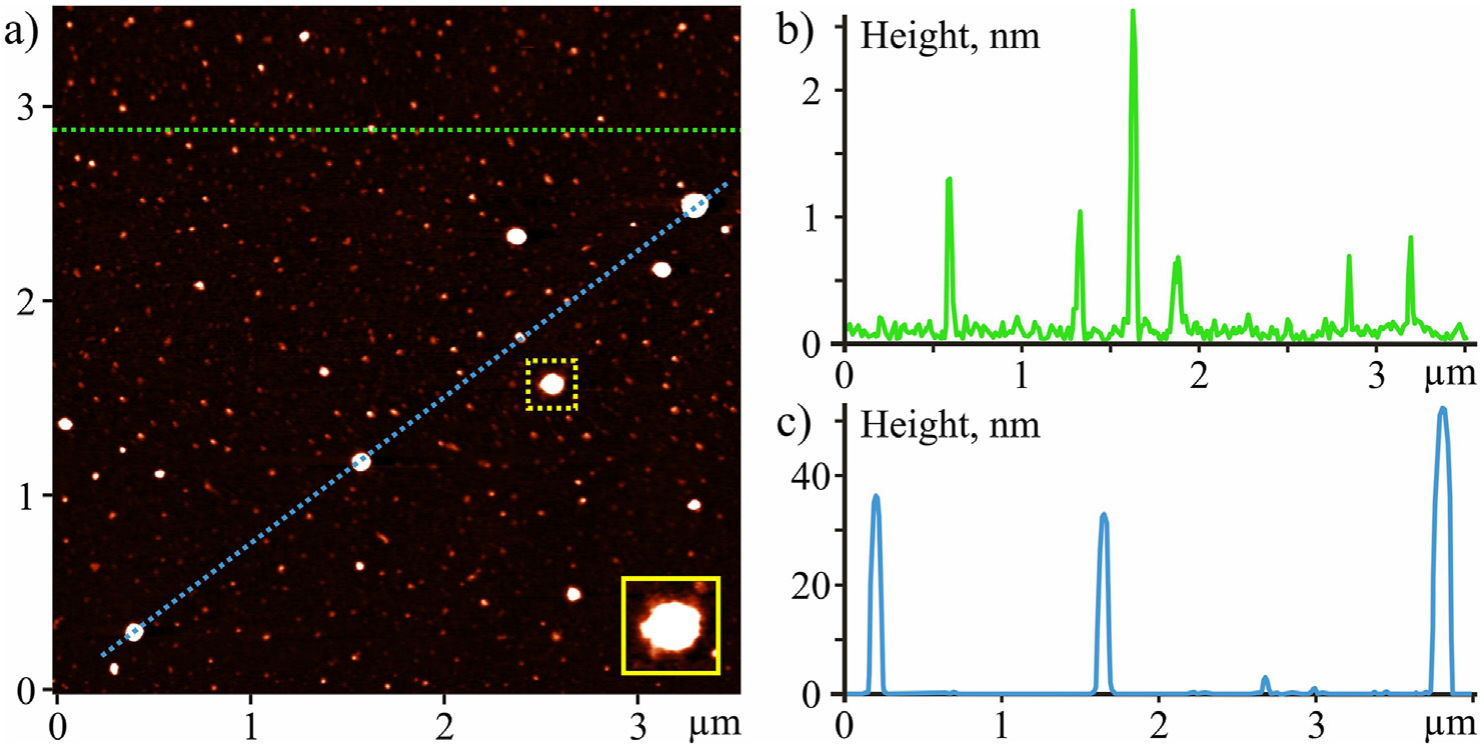
AFM image of the C_60_ fullerene layer deposited from C_60_FAS (0.15 mg mL^−1^) on the surface of mica (a), and its Z-section along the lines indicated in the images (b and c).

Clear AFM images of C_60_ fullerene were obtained (Figure 1a), which indicates the high chemical purity of both the material and the solvent, and the measurement conditions. The images show chaotically placed objects, which in shape and size can be divided into two groups. The first of them includes point objects up to 10 nm high, among which the maximum number was with heights in the range of 0.7–3 nm (Figure 1b). The height of the smallest of them (0.7 ± 0.2 nm) agrees well with the molecular diameter of C_60_, which allowed us to identify them as individual molecules. Larger objects correspond to C_60_ fullerene bulk clusters. They had symmetrical bell-shaped profiles with sharp maxima in the middle. This is a sign that the aggregation of some molecules into clusters took place in C_60_FAS before they were applied to the substrate.

The second group includes objects with a height of 10–100 nm of irregular shape (Figure 1c and selected fragments in Figure 1a). They are characterized by fuzzy contours or the presence of protrusions with a thickness of one monolayer at the edges. This is characteristic of the self-assembly of molecules on the substrate surface during deposition from solution to form bulk aggregates according to the known Volmer – Weber islet growth mechanism.

### 3.2 Analysis of muscle contraction force

**Figure 2:**
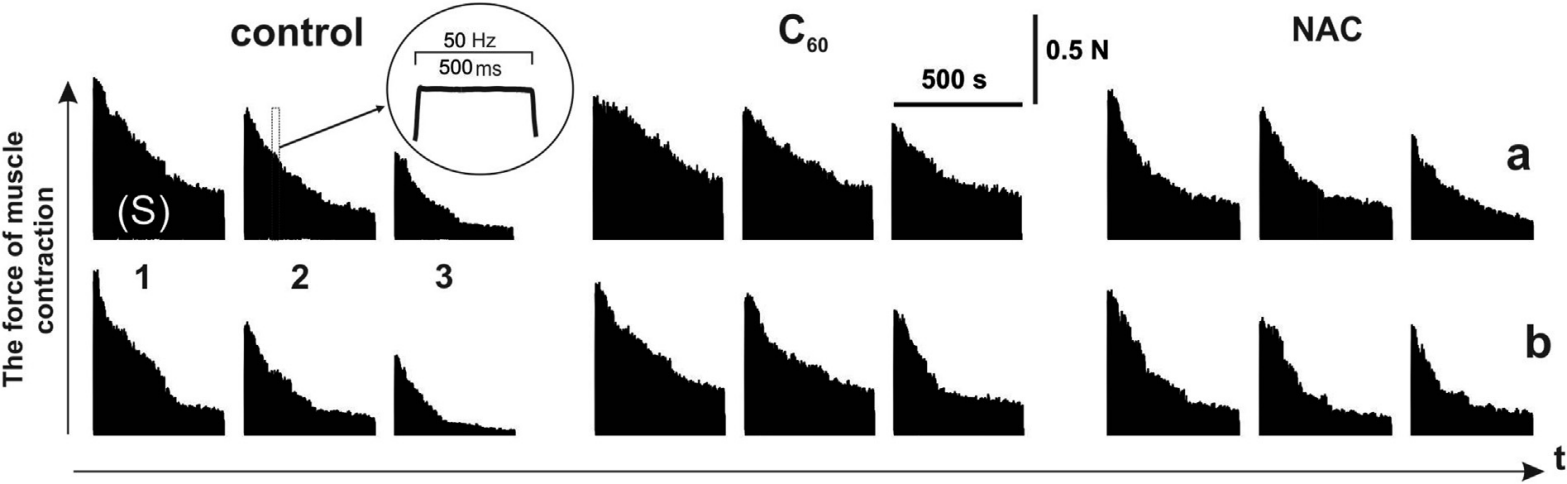
Contraction force of rat *m. soleus* (a) and m. gastrocnemius (b) after application of 50 Hz stimulation for 5 s with three successive pools: 1, 2, 3 - consecutive stimulation pools of 500 s duration each with 5 min relaxation between them; control - native muscle; C_60_ - muscle mechanograms 1 h after C_60_FAS injection; NAC - muscle mechanograms 1 h after N-acetylcysteine injection; S - integrated muscle power (calculated area under the force curve).

Recording the contractions force of *m. soleus* and *m. gastrocnemius* by stimulation pools (Figure 2a and 2b) has revealed a significant difference in the development of their fatigue processes and was 31 ± 3%, 39 ± 1%, and 47 ± 4% at 1,2 and 3 stimulation pools, respectively. These results confirm the data [1] about the greater sensitivity of fast muscle fibers to the development of fatigue.

**Figure 3:**
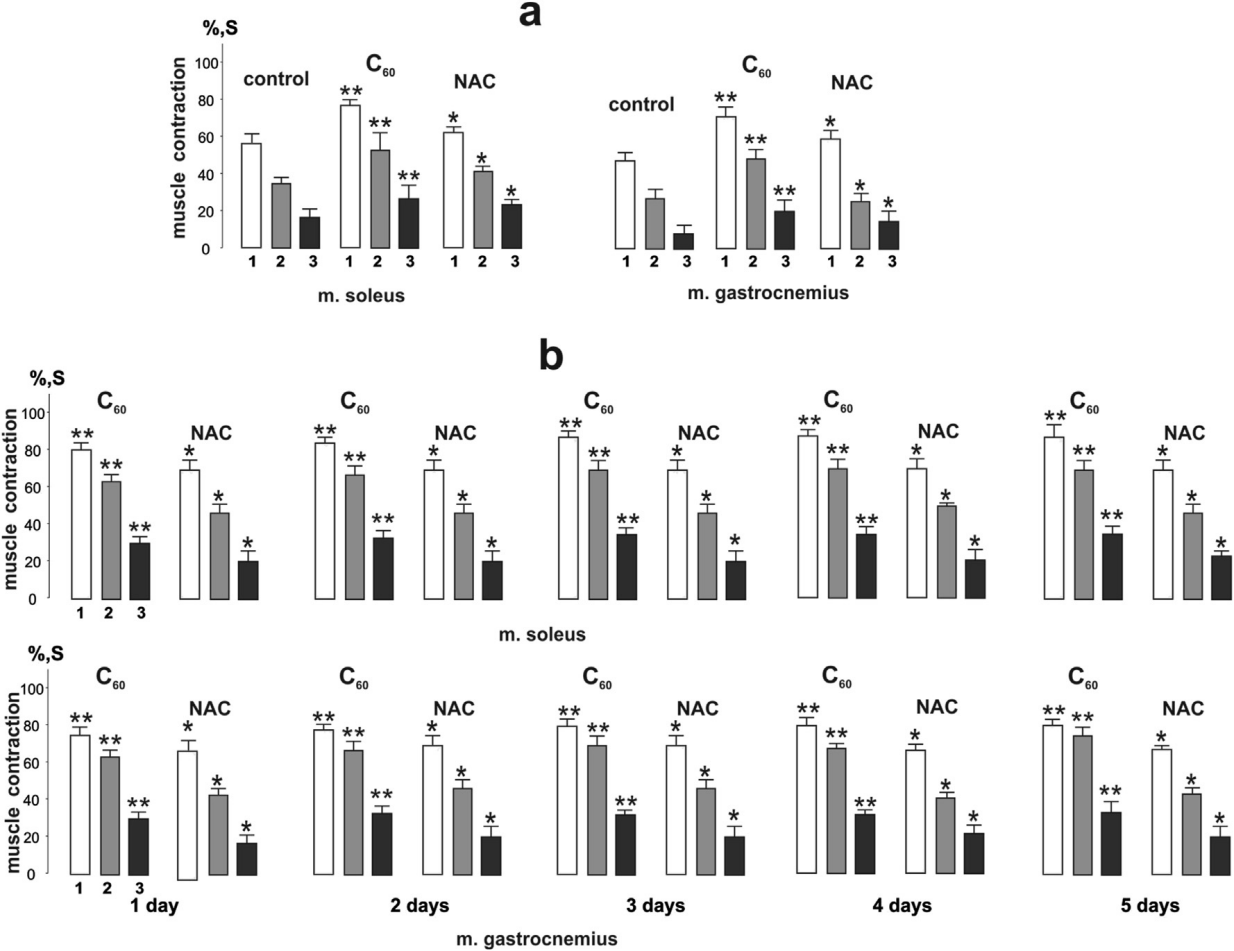
Integrated rat *m. soleus* and *m. gastrocnemius* power (S), presented as a percentage of maximum values after application of 50 Hz stimulation for 5 s in three successive pools (1, 2, 3) lasting 500 s each with 5 min of relaxation between them: muscle integrated power values 1 h after C_60_FAS (C_60_) and NAC administration (a); muscle integrated power values 2 h after C_60_FAS (C_60_) and NAC administration during 1st, 2nd, 3rd, 4th and 5th days (b). ∗*p* < 0.05 compared to control (fatigue) value; ∗∗*p* < 0.05 compared to value in NAC group; *n* = 7 in each group.

Mechanograms obtained after 1 h of C_60_ fullerene administration and NAC (Figure 3a) demonstrated a more pronounced effect of C_60_ fullerene, being 19 ± 1%, 17 ± 1%, and 24 ± 3% greater than for NAC in *m. soleus* and 24 ± 2%, 26 ± 1%, and 29 ± 3% greater than in *m. gastrocnemius* at 1,2 and 3 stimulation pools, respectively.

The analysis of mechanograms obtained 2 h after injections of the drugs (Figure 3b) revealed a further slight increase in the effect of C_60_ fullerene, namely by (7–9) ± 1% in *m. gastrocnemius* and (5–6) ± 1% in *m. soleus* at three studied stimulation pools. Note that the effect of NAC did not change significantly. On this basis, in the future, we analyzed the long-term use of the studied drugs 2 h after their administration.

The analysis of the values of the integrated muscle power 2 h after drug administration on the 1st, 2nd, 3rd, 4th, and 5th days showed an increase in the effect of C_60_ fullerene on the 4th day. The growth of positive effect was 8 ± 1%, 6 ± 1%, and 4 ± 1% on the 5th day in *m. soleus* and 14 ± 1%, 12 ± 1% and 6 ± 1% in *m. gastrocnemius* at 1,2 and 3 stimulation pools, respectively. It should also be noted that the use of NAC did not reveal a significant increase in efficacy already on the 2nd day of its use.

Thus, the most significant effects were observed on the 3rd day of drug use. However, further application of NAC did not cause significant changes in muscle fatigue processes. An increase in the protective effect of C_60_ occurred within 4 days (the difference between 4 and 5 days did not exceed (3–5)%) and exceeded the effect of NAC by (32–34)%.

In summary, the data obtained for the force response of the muscle against the background of the development of muscle fatigue indicate that the administration of C_60_ fullerenes (for at least 4 days) reduces the severity of pathological processes by (35–45)% in slow muscle and by (60–65)% in fast muscle. This confirms that the use of C_60_FAC in a low dose leads to a decrease in the recovery time of muscle contraction force and an increase in the time of its function [27, 40].

### 3.3 Analysis of biochemical parameters in muscle tissues

**Figure 4:**
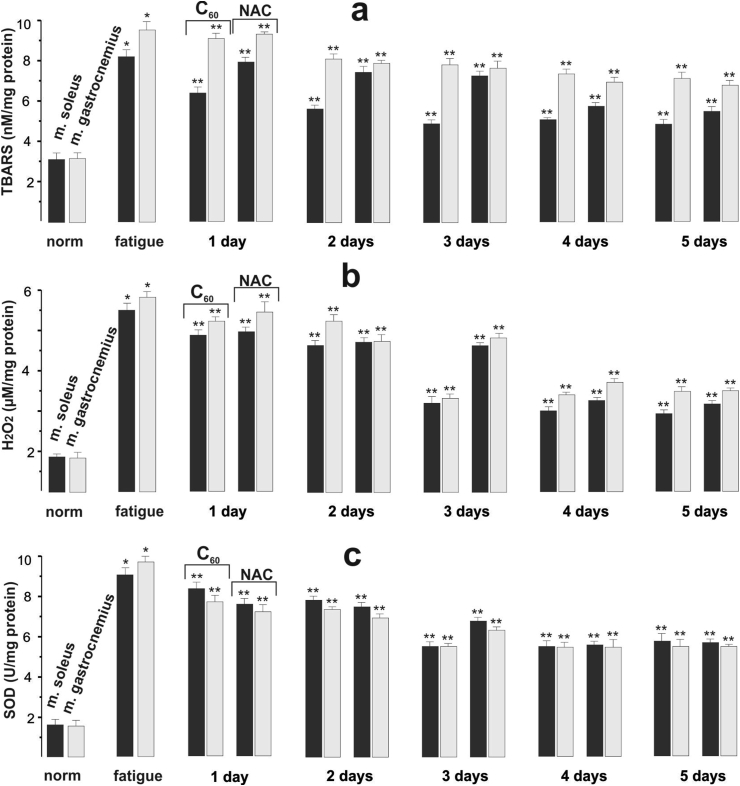
Indices of pro- and antioxidant balance (TBARS (a), H_2_O_2_ (b), and SOD (c)) in the studied rat muscle tissues after application of three-component stimulation and administration of C_60_FAS (C_60_) and NAC for 1,2,3,4 and 5 days. ∗*p* < 0.05 relative to the intact (norm) group; ∗∗*p* < 0.05 relative to the control (fatigue) group; *n* = 7 in each group.

It was found that long-term administration (for 5 days) of C_60_ fullerene led to a gradual decrease in TBARS and H_2_O_2_ content in both *m. soleus* and *m. gastrocnemius* of rats (Figure 4a,b).

Thus, on day 5, the content of secondary LPO products and hydrogen peroxide in *m. gastrocnemius* decreased by 28% and 44%, respectively, and in *m. soleus* by 29% and 40%, respectively, compared with the first day of the experiment. The observed inhibition of excessive accumulation of LPO products and oxygen derivative H_2_O_2_ in both muscle types may be mediated by the antiradical properties of C_60_ fullerene. The results confirm that C_60_ fullerenes can penetrate through plasma membranes and accumulate in particular tissues, including muscles, without signs of damage [19, 41].

Antioxidant defense enzymes such as SOD, CAT, and GP_x_ play a key role in the mechanisms of regulation of free radicals and peroxide processes. SOD is the most powerful natural antioxidant and the enzyme of the first link of antioxidant protection, which carries out the dismutation reaction of superoxide anion radicals and converts them into less reactive hydrogen peroxide molecules [42].

Previous studies have shown [43] that SOD activity and protein accumulation in rat skeletal muscles increased during exercise without significant changes in mRNA expression. At the same time, SOD activity increased predominantly in oxidative muscles with a high content of type I and IIa fibers [44]. In our experiment (Figure 4c), during electrical stimulation of muscles against the background of C_60_ fullerene application, a gradual decrease in SOD activity was found in *m. gastrocnemius* (by 13%) on day 5, while in *m. soleus* SOD activity increased from day 3–4 (by 27% and 21%, respectively), and on day 5 only tended to increase relative to the first day of the experiment.

**Figure 5:**
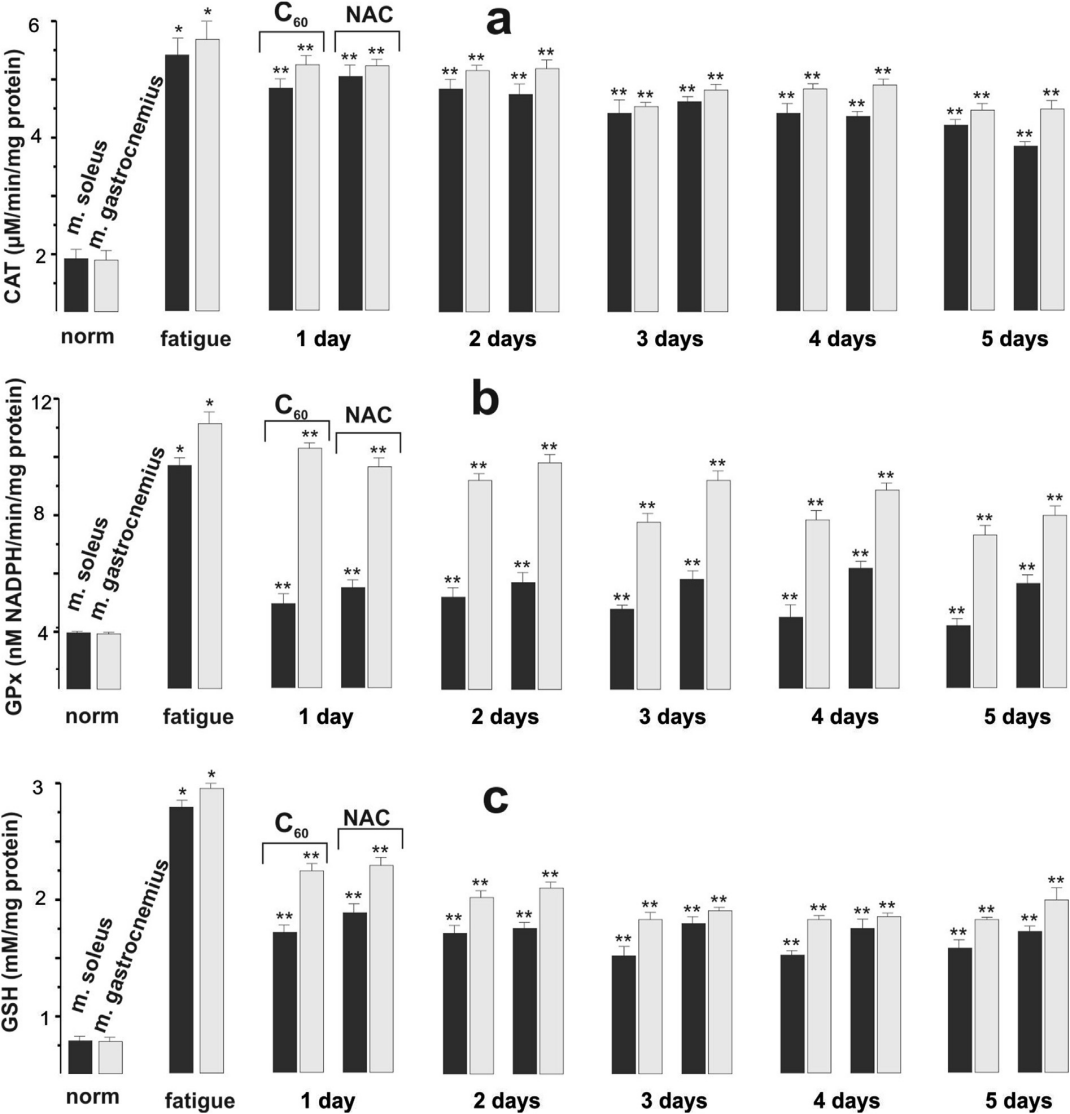
Indices of antioxidant defense (CAT (a), GPx (b), and GSH (c)) in the studied rat muscle tissues after the initiation of three-component stimulation and administration of C_60_FAC (C_60_) and NAC for 1,2,3,4 and 5 days. ∗*p* < 0.05 relative to the intact (norm) group; ∗∗*p* < 0.05 relative to the control (fatigue) group; *n* = 7 in each group.

Physical exercise against the background of long-term administration of C_60_ fullerene did not lead to significant changes in CAT activity in both *m. gastrocnemius* and *m. soleus* during the 5 days of the experiment (Figure 5a). CAT activity significantly decreased in *m. gastrocnemius*, by 33% on day 5 of the experiment compared with day 1, while in *m. soleus* the activity of this enzyme only tended to decrease. Despite a considerable number of studies, the data on the activity of such endogenous antioxidants as CAT and GP_x_ during intensive physical activity of skeletal muscle remain contradictory. It was found that chronic exercise does not change CAT activity in slow muscles, but decreases it in fast muscles [45]. CAT activity has also been shown to decrease in both oxidative and glycolytic muscle fiber types [46]. In contrast, other studies have shown an increase in CAT activity [47].

Cellular mechanisms of antioxidant protection are also associated with the functioning of a powerful glutathione link [13]. Along with antiradical enzymes, the glutathione system is one of the active components of the body’s antioxidant defense, which plays a significant role in the attenuation of the pathological process during muscle fatigue, since it not only prevents the free-radical reactions but also provides effective elimination of the final metabolites of LPO. The protective functions of GSH during oxidative stress are determined by the ability to catalyze the cleavage of hydrogen peroxide and fatty acid hydroperoxide with GSH [48].

In our study, after administration of C_60_ fullerenes against the background of fatigue development for 5 days, GP_x_ activity in *m. soleus* gradually decreased by 15%. At the same time, in *m. gastrocnemius* the activity of this anti-peroxide enzyme during the same period had a tendency only to decrease (Figure 5b). It is important to note that GP_x_ activity in *m. soleus* was three times higher than in *m. gastrocnemius*. This coincides with previous studies showing a significant increase in GP_x_ activity in oxidative muscle fibers under physical strain [45, 49]. At the same time, the mRNA level of GP_x_ expression in these muscles corresponded to the degree of activity of this enzyme [50].

The tendency established in our experiments to decrease the activity of antiradical (SOD) and anti-peroxide (CAT and GP_x_) enzymes when using C_60_ fullerene in the development of muscle fatigue confirm the slowdown of oxidative processes in skeletal muscles of both types. Such dynamics of the activity of the above enzymes can testify to the efficiency of dismutation processes with a decrease in the level of aggressive superoxide radical, as well as the processes of elimination of peroxide compounds in rat muscle fibers [12].

Glutathione belongs to the main links of antioxidant protection, participating in the detoxification of xenobiotics and toxic metabolic products, the process of apoptosis, affects enzyme activity and nucleic acid biosynthesis, and regulates eicosanoids, and prostaglandins exchange [13, 48]. The use of C_60_ fullerene against the background of muscle fatigue caused the GSH content in *m. gastrocnemius* and *m. soleus* to increase by 51% and 61%, respectively, on the 5th day of the experiment compared to the 1st day (Figure 5c). This agrees with previous studies [51], which found that C_60_ fullerene can influence the processes of glutathione synthesis and metabolism in different tissues, including muscle, under different pathological conditions by modulating the Nrf2/ARE-antioxidant pathway. We also showed that C_60_ fullerenes prevent mitochondrial dysfunction by restoring the activity of enzymes of mitochondrial complexes, as well as inhibiting mitochondrial-dependent apoptosis by limiting mitochondrial translocation of the p53 protein and increasing the expression of the Bcl-2 protein [52]. Consequently, the use of C_60_ fullerene led to an increase in the efficiency of the antioxidant defense system due to an increase in the GSH content in both slow and fast muscles, thereby increasing their resistance to physical activity.

If we compare the dynamics of changes in pro- and antioxidant homeostasis parameters between fast and slow muscles, it should be noted that the intensity of free-radical processes in these muscles is determined primarily by the peculiarities of their metabolism, functional load, and the level of antioxidant protection system [16]. It is known that during physical exercise the activity of SOD, GP_x_, CAT enzymes, as well as GSH content, are the highest in oxidative muscles (type I and type IIa) that have an increased blood supply, much myoglobin, a large number of mitochondria, and energy supply mainly due to oxidative phosphorylation processes [53]. In our study, when C_60_ fullerene was applied during the development of muscle fatigue, the intensity of oxidative processes as well as the level of the antioxidant defense system in the fast and slow muscles of rats differed from each other. TBARS and H_2_O_2_ content in slow muscles were (70–71)% and (29–41)% higher than in fast muscles, respectively. Moreover, in slow muscles, the activity of anti-peroxide enzymes and GSH content exceeded those in fast muscles, and the extent of this increase was different. Thus, in slow muscles, CAT activity and GSH content were 8 and 6% higher, respectively, than in fast muscles. In slow muscles, GP_x_ activity was three times greater than in fast muscles. This tendency of the flow of antioxidant processes coincides with the data [50] regarding the difference in the activity and levels of mRNA expression of CAT and GP_x_ between fast and slow muscles at rest and after heavy exercise. However, our experiments showed that SOD activity in fast muscles was 35% higher than in slow muscles when C_60_ fullerene was applied.

Thus, the long-term use of C_60_ fullerene slows down the course of oxidative stress in fast and slow muscles by maintaining the balance between pro-oxidants and the antioxidant defense system, which prevents the negative effects of ROS on cellular and subcellular structures while muscle fatigue development in rats.

According to the literature data, NAC as a known glutathione precursor can neutralize free radicals and thus exhibit antiradical and antioxidant properties [54]. In addition, numerous studies have shown that the use of NAC removes muscle fatigue during submaximal exercise in humans, including electrical muscle stimulation [55]. Therefore, in our study, we used NAC as a comparison drug. NAC administration during electrostimulation of the rat muscles for 5 days also caused a decrease in TBARS and H_2_O_2_ content in fast muscles by 42% and 44%, respectively, and in slow muscles by 38% and 48%, respectively, compared with the first day of the experiment. The obtained data coincide with the results of other authors who showed the effectiveness of NAC in the elimination of ROS [56]. NAC application in the simulation of muscle fatigue caused a 29% decrease in CAT activity in fast muscles on day 5, while in slow muscles we registered only a decreasing tendency. SOD activity did not undergo significant changes in the two types of muscles studied throughout the experiment. At the same time, GSH content and GP_x_ activity in the fast muscles increased by 30% and 43%, respectively, and in the slow muscles by 21% and 34%, respectively, on day 5.

A comparative analysis of oxidative stress markers and indicators of the state of antioxidant defense systems showed that the protective effect of C_60_ fullerene was higher on the first day compared to NAC by (5–10)% in fast and slow muscles, increased to (20–35)% after 3 days of drug use and additionally increased by (8–9)% to 5th day.

## 4 Conclusions

The analysis of the obtained data showed positive dynamics of changes in the force of muscle contraction, markers of oxidative stress, and indicators of the state of antioxidant protection systems in fast and slow muscles of rats with the intraabdominal injection of C_60_ fullerene and NAC at low doses of 1 and 150 mg kg^−1^, respectively, as potential correctors of the effects of muscle fatigue. So, the effect of C_60_ fullerene on fast muscles was (15–17)% more effective than on slow muscles in terms of muscle force response against the background of fatigue development 1 h after C_60_ fullerene administration. Analysis of mechanograms 2 h after drug injection revealed a further slight increase in the effect of C_60_ fullerene, namely by (7–9)% in *m. gastrocnemius* and (5–6)% in *m. soleus*. The maximum effect was observed on the 1st day of the experiment. The increase C_60_ fullerene effect occurred within 4 days (the difference between the 4th and 5th day did not exceed (3–5)%) and exceeded the effect of NAC by (32–34)% overall.

The analysis of biochemical parameters in rat muscle tissues against the background of induced muscle fatigue showed that long-term application of C_60_ fullerene (for 5 days) slows down the course of oxidative stress by (10–30)% in fast muscles and by (5–20)% in slow muscles due to maintaining a balance between pro-oxidants and antioxidant defense system. The maximum decrease in biochemical markers was recorded after 3 daily administrations of drugs. At the same time, the protective effect of C_60_ fullerene was higher compared to NAC by (20–35)%, and this difference additionally increased by (8–9)% to the 5th day.

Thus, the above data indicate the prospect of using water-soluble C_60_ fullerenes, whose antioxidant effect exceeds the effect of the known compound NAC, as potential protective nanoagents to improve the efficiency of skeletal muscle function by modifying the ROS-dependent mechanisms that play an important role in the development of fatigue processes.

## Declarations

### Author contribution statement

Dmytro Nozdrenko, Kateryna Bogutska, Olga Gonchar, Svitlana Prylutska, Daria Franskevych, Yuriy Prylutskyy, Bohdan Hromovyk, Peter Scharff and Uwe Ritter: Conceived and designed the experiments, Performed the experiments, Analysed and interpreted the data, Contributed reagents, materials, analysis tools or data, Wrote the paper.

### Funding statement

Yuriy Prylutskyy was supported by 10.13039/501100007684Ministry of Education and Science of Ukraine [№ 21БП018-01Р].

### Data availability statement

Data will be made available on request.

### Declaration of interest’s statement

The authors declare no competing interests.

### Additional information

No additional information is available for this paper.
